# Patient global assessment in measuring disease activity in rheumatoid arthritis: a review of the literature

**DOI:** 10.1186/s13075-016-1151-6

**Published:** 2016-10-28

**Authors:** Elena Nikiphorou, Helga Radner, Katerina Chatzidionysiou, Carole Desthieux, Codruta Zabalan, Yvonne van Eijk-Hustings, William G. Dixon, Kimme L. Hyrich, Johan Askling, Laure Gossec

**Affiliations:** 1 Department of Rheumatology, Whittington Hospital NHS Trust, London, UK; 2 Medical University Vienna, Vienna, Austria; 3 Rheumatology Department, Karolinska University Hospital and Department of Medicine, Karolinska Institute, Stockholm, Sweden; 4 Sorbonne Universités, UPMC University Paris 06, Institut Pierre Louis d’Epidémiologie et de Santé Publique, GRC-UPMC 08 (EEMOIS); AP-HP, Pitié Salpêtrière Hospital, Department of Rheumatology, Paris, France; 5 Sorbonne Universités, UPMC University Paris 06, Institut Pierre Louis d’Epidémiologie et de Santé Publique, GRC-UPMC 08 (EEMOIS), Paris, France; 6 Department of Patient & Care, Department of Rheumatology, Maastricht, the Netherlands; 7 Romanian League against Rheumatism, Bucharest, Romania; 8 Department of Patient & Care, Maastricht, The Netherlands; 9 Department of Rheumatology, Maastricht, The Netherlands; 10 Arthritis Research UK Centre for Epidemiology, Manchester Academic Health Science Centre, University of Manchester, Manchester, UK; 11 NIHR Manchester Musculoskeletal Biomedical Research Unit, Central Manchester NHS Foundation Trust, and Manchester Academic Health Science Centre, Manchester, UK

**Keywords:** Rheumatoid arthritis, Patient global assessment, Discordance

## Abstract

Patient-reported outcomes (PROs) reflect the patient’s perspective and are used in rheumatoid arthritis (RA) routine clinical practice. Patient global assessment (PGA) is one of the most widely used PROs in RA practice and research and is included in several composite scores such as the 28-joint Disease Activity Score (DAS28). PGA is often assessed by a single question with a 0–10 or 0–100 response. The content can vary and relates either to global health (e.g., how is your health overall) or to disease activity (e.g., how active is your arthritis). The wordings used as anchors, i.e., for the score of 0, 10, or 100 according to the scale used, and the timing (i.e., this day or this week) also vary. The different possible ways of measuring PGA translate into variations in its interpretation and reporting and may impact on measures of disease activity and consequently achievement of treat-to-target goals. Furthermore, although PGA is associated with objective measures of disease activity, it is also associated with other aspects of health, such as psychological distress or comorbidities, which leads to situations of discordance between objective RA assessments and PGA. Focusing on the role of PGA, its use and interpretation in RA, this review explores its validity and correlations with other disease measures and its overall value for research and routine clinical practice.

## Background

Patient-reported outcomes (PROs) are increasingly recognized for their value in providing the patient’s perspective on aspects of their condition or their overall health status. Their incorporation into clinical practice and in research in rheumatoid arthritis (RA) is widely supported by international organizations and professional bodies [1, 2], including the European Patients’ Academy on Therapeutic Innovations (EUPATI; http://www.patientsacademy.eu/index.php/en/) and the Patient-Centered Outcomes Research Institute (PCORI; http://www.pcori.org/research-results) in the United States, as well as regulatory agencies such as the Food and Drug Administration (http://www.fda.gov/) and the European Medicines Agency (http://www.ema.europa.eu/ema/), all of whom recognize the patient’s unique position in providing direct feedback on their disease.

Patient global assessment (PGA) is one of the most widely reported PROs in RA. The considerable burden of RA on the individual is related to both inflammation and damage but also to broader aspects of disease, including psychological and societal impact. The use of PROs like the widely used Health Assessment Questionnaire (HAQ) or the PGA allows a more holistic assessment of disease beyond objective measures of inflammation or structural damage, such as acute phase reactants or radiographic damage. Experts from the American College of Rheumatology (ACR), the European League Against Rheumatism (EULAR), and the Outcome Measures in Rheumatology Clinical Trials (OMERACT) have endorsed a “core set” of data for use in RA clinical trials which includes PGA [3, 4]. In recent randomized controlled trials and observational studies in RA, PGA has been reported in 49 % of studies, making it the second most frequently collected PRO after physical function (68 %) [5]. PGA is also incorporated into several of the major outcome and disease activity scores in RA, often as the only PRO: these include the ACR/EULAR remission criteria, the 28-joint count Disease Activity Score (DAS28), the Simplified Disease Activity Index (SDAI), the Clinical Disease Activity Index (CDAI), and the Routine Assessment of Patient Index Data (RAPID3).

However, the use of PGA in RA presents many challenges and limitations. The several possible ways of measuring PGA, including the intended assessment or underlying concept (i.e., global health versus disease activity) and variations in wording/phrasing and time period assessed may lead to differences in interpretation of PGA. Discordance with objective RA measures is also an issue that needs to be addressed. The latter is particularly important in the context of treating to target aiming for remission and shared decision-making [6, 7]. What the different formulations of PGA are, their impact, and justifications for their use remain to be clarified.

To provide readers with a complete overview regarding PGA in RA, a review of the literature was undertaken based on a hierarchical literature search including hand searches and expert opinion searches covering key publications in the field. The objectives were to explore the value of PGA as an outcome measure in RA, focusing on its psychometric properties (feasibility, validity, reliability, and sensitivity to change). Specifically, this review discusses the validity and impact of different wordings/phrasings and time period assessed as part of PGA on patients’ assessment of disease, as well as discordance between physician global assessment and PGA.

## Description and practical application

### Concepts behind PGA

PGA was developed in the late 1970s and was initially designed for the measurement of self-assessed *pain* in RA [8], although it has since been used to evaluate RA more globally. It is interesting to note that the way PGA is used in clinical practice covers, in fact, two very different concepts, one related to *global health* and the other to *overall disease activity*. They are both usually used under the heading of PGA without further specification for which is being assessed.

### PGA wording and phrasing

It is well-recognized that the wording/specific phrasing used for PROs may result in a varied response [9–12]. In the case of PGA, its exact wording/phrasing was not specified when developed; however, it was suggested that it could be used for two main purposes—either a patient assessment of global health or of disease activity—stemming from the two basic concepts (Table 1) [11, 13]. Over the past years many different wordings/phrasings of PGA have been formulated, covering variations of these two concepts [14–18]. Furthermore, anchor wordings may also vary, e.g., words used to describe the right end of the score (corresponding to scores of 10 or 100) from “worst possible” to “most active” to “very active”, for example.

**Table 1:** Different concepts covered by PGA and examples of different types of wording used

Concept	Attribution to RA	Example question	Reference period
Disease activity	Related to arthritis	“Considering all the ways your arthritis has affected you, how active do you feel your arthritis is …” “Considering the tenderness, pain, and swelling of joints, how active is your rheumatoid arthritis ....” “In general, how active has your rheumatic condition been?” “How active do you consider your arthritis?” “In terms of joint tenderness (i.e., joint pain associated with light touch) and joint swelling (i.e., joint enlargement due to inflammation), how active would you say your rheumatic condition is today?’	Today Over the past 2 days Last week Last month Unspecified time period
	Overall	“How do you estimate your disease activity ..?”	
Global health	Related to arthritis	“Considering all the ways your arthritis has affected you, how would you say your health is …” “Considering all the ways in which your illness affects you at this time, please make a mark below to show how you are doing” “How has your arthritis affected you today?” “Considering all the ways your arthritis affects you, rate how well you are doing on the following scale…”	
	Overall	“Considering all the ways in which illness and health conditions may affect you at this time, please make a mark below to show how you are doing” “In general how would you say your health is”	

Although the wording/phrasing of PGA remains unstandardized to date, the ACR/EULAR remission criteria do specifically propose the following phrasing related to disease activity: “Considering all the ways your arthritis has affected you, how do you feel your arthritis is today?” [19].

### PGA reference period

Aside from different wording/phrasing used for the question stem, the reference periods to describe the time component (i.e., the period of recall the patient should refer to when answering the question) can also vary (Table 1). As we will see in this review, the different formulations of PGA lead to differences in interpretation.

In the context of a EULAR taskforce to standardize data collection across registries, in 2015 we contacted registries and cohorts across Europe to explore outcomes being assessed: 52 out of 67 (78 %) registries were collecting some form of PGA [20]. The versions of the PGA used varied with regard to the concept, wording/phrasing, and reference period used. More recently, a smaller pilot survey in 2016 (unpublished data) indicated that 6/16 (38 %) cohorts were assessing disease activity-PGA (either related to RA or not specifically related to RA) whereas 6/16 (38 %) were assessing global health PGA and 4/16 (25 %) were assessing both concepts. Some wordings as translated by the investigators are shown in Table 1. With regard to the reference period, 41 % reported “today” as being the time reference used, with the second most common being “last week” (35 %).

### PGA scoring

Depending on the type of score used, the PGA can range from 0–100 mm, although is often reported from 0–10 cm. Higher scores represent a higher level of disease activity or a worse global health. The proposed definition of “low global assessment” is ≤2.0 (scale 0–10) [21].

PGA may be scored using a numeric rating scale (NRS), a verbally administered NRS, or a visual analogue scale (VAS). The PGA-VAS is classically anchored on an unnumbered 10-cm/100-mm horizontal line but may also be administered as a vertical VAS. The VAS may be anchored at the ends (e.g., with defined adjectives at the ends such as best versus worst) or open. Sometimes the PGA is presented with tick marks at periodic intervals or as a VAS consisting of 21 circles at 0.5-mm intervals, the latter shown to be similar to a classic 10/100 scale [22]. In practice, sometimes these exact definitions are not followed (e.g., the line is not 10 cm long or there are not 21 circles evenly spaced) and these technical difficulties may hamper the use of PGA (as is the case with other PROs). A Likert-style scale may also be used, though its metric properties are different.

A study comparing responses to a global health VAS presented both as a 10/100 cm horizontal scale with no incremental markers and as a vertical 20/100 cm VAS with 1-cm markers concluded that different presentation of scales, order effect, and incremental markers can affect scoring [14]. Another study comparing different scaling of PGA revealed similar construct validity for VAS and NRS but higher sensitivity to change of VAS [23]. Although some differences can be seen in scoring methods for PGA, all methods appear at this point similarly valid. We would, however, recommend the use of either an unnumbered horizontal VAS or a numbered horizontal NRS since these formats are the most usual and most used.

## Psychometric properties of PGA

The main strengths and weaknesses of PGA are summarized in Table 2. Below, we review each psychometric property of PGA.

**Table 2:** Major strengths and weaknesses of PGA in RA

Strengths	Weaknesses
Practical and feasible to collect: much more easily collected than joint counts, acute phase reactants, or radiographic damage (simple, single-item tool)	Heterogeneity in concept (i.e., global health versus disease activity) and attribution to RA or other co-existing health conditions and wording/phrasing, all leading to possible heterogeneity in the responses
No cost, non-invasive and self-administered	Heterogeneous phrasing of the time-frame (today, last week, etc.) applied to PGA
May summarize all aspects of disease important to the patient (face validity)	Very broad concept leading to interpretation difficulties
Practical and feasible to interpret: easy to score, incorporate in composite scores, and analyze	Difficulties of interpretation due to uncertainty regarding attribution to permanent damage related to RA compared to inflammation and disease activity
Good test–retest reliability	Difficulties of interpretation due to uncertainty regarding attribution to RA versus non-RA disease, including psychological distress and comorbidities
Good sensitivity to change in clinical trials	May be influenced by patient education level
Discordance between PGA and physician assessment: brings in additional information	Discordance between PGA and physician assessment: what impact on decision making?

### Feasibility

Like other PROs, including, for example, the HAQ, PGA is a very feasible measure. PGA is administered as a simple, single-item (with no subscale), patient-completed question measuring the overall way RA affects the patient and/or disease activity at a specific point in time. There is no cost attached to it, it is practical, and can be self-administered. The single question takes only a few seconds to ask, making it feasible in routine clinical settings but also as an end-point in clinical trials in RA and is one of the main strengths of the PGA, making it one of the most frequently reported domains across published RA studies [6].

### Face validity

PGA is a global, “gestalt” measure of disease which appears to encompass many aspects of disease which are important for patients. Physicians’ assessment of RA disease activity is mainly driven by objective criteria, i.e., tender/swollen joint counts and level of inflammation, whereas it seems patients place more focus on overall well-being, levels of pain, and health-related quality of life [24]. The latter, in particular, seems to have the greatest relevance and meaning to patients. However, it is often difficult to capture health-related quality of life with simple questionnaires. In this sense, PGA appears of interest since it may summarize in one simple measure many aspects of disease and health which are important to patients.

Although PGA has high face validity (Table 2), it does present some challenges. A major point is the patient’s interpretation of the PGA, both depending on the concept (i.e., global health versus disease activity) and on the patient’s individual comprehension of this broad question. For both concepts behind PGA, a criticism is that the response to the question may both reflect a broad understanding of the patient’s health and also be influenced by a number of factors, making it difficult to discern what aspect of disease contributes to the overall score. Structural damage (related to disease duration) and other aspects of patients’ lives (such as comorbidities or psychological distress) may have an impact on the scoring of PGA [1].

In both cases of PGA (i.e., global health versus disease activity), interpretation of the question by the patient may depend on duration of disease through a “response shift” (i.e., a change in the meaning of a patient’s self-evaluation) resulting from a better knowledge of symptoms and changes in patient expectations [25]. In the current biologic era, however, cumulative damage is considerably lower despite longer disease duration and therefore the effect and meaning of a response shift may have a different interpretation. Differences in patients’ perceptions regarding internal standards, values, or conceptualization of health-related quality of life can result in “ambiguous” or “paradoxical” findings [25]. For example, patients with long-standing disability may report a good or high quality of life (despite what externally might appear paradoxically untrue) due to several factors, such as acceptance and the opportunity to adjust and achieve stability through several transition phases while living with disability. All these factors need to be taken into account when interpreting individuals’ PGA scores.

### Reliability

Data on the reliability of PGA, which refers to the reproducibility in a test–retest setting, are reassuring [26–28]. Studies have shown the PGA intraclass correlation coefficient (as a measure of test–retest reliability) to be generally acceptable to high, though lower than ones noted for physician global assessment [29, 30]. The data available in the literature do not allow us to directly compare reliability of PGA–global health versus PGA–disease activity; although when tested separately, both appear to have acceptable reliability.

### Sensitivity to change

Sensitivity to change indicates that the measure will improve when the underlying conceptual framework (here, either global health or disease activity) improves. PGA has been shown to be sensitive to change, which makes it a very useful clinical measure in assessing RA, particularly in clinical trials. PGA detects improvement after active treatment better than, for example, tender joint count [31]. Table 3 summarizes key findings in this area. Furthermore, PGA has been shown to discriminate active treatment from placebo in randomized controlled trials, with treatment-associated changes being congruent with measures of inflammation, suggesting close reflection of other criteria related to the RA process, such as joint counts [32].

**Table 3:** Sensitivity of PGA to change in disease activity and comparison with other measures of disease

Study group	Study details	Main findings
Kaneko et al. 2014 [46]	Prospective study Newly diagnosed RA 75 patients Discordance between PGA and EGA	PGA more sensitive for indicating progressive joint destruction and functional impairment when compared with EGA Discrepancy directed toward a worse assessment by patients
Pope et al. 2009 [52]	Prospective study of a large clinical practice 225 RA patients MID estimates for: (1) HAQ-DI improvement and worsening using PGA anchor; and (2) pain using a patient-reported pain anchor.	MID scores for HAQ-DI in clinical practice were smaller than those seen in clinical trials MID scores were influenced by baseline HRQOL scores and may be influenced by disease duration MID changes were different for worsening (usually needing a larger value) than for improving MID for deterioration was much less than for improvement in patients with more pain and impairment in physical function
Wells et al. 2008 [31]	Randomized controlled trial comparing abatacept (n = 258) with placebo (n = 133) in anti-TNF poor respondents (ATTAIN study) Evaluation of the responsiveness of PROs in RA patients	PGA had larger relative percentage improvement with treatment (24 %) than the generic quality of life outcomes SF-36 domains and component scores (range 8–21 %) PGA was more efficient than TJC in detecting a treatment effect PGA was found to be in close proximity to the ESR, physician global assessment and the PROs pain assessment, HAQ, bodily pain and physical component score in terms of the standardized response means
Lassere et al. 2001 [53]	Literature review on reliability for different classes of RA measures	The SDD for the PGA (as well as for SJC, TJC, and pain) was found to be large and it had poor reliability compared to multi-item measures of physical and psychological function and radiologic measures
Ward 1994 [54]	Prospective study 24 RA patients Determination of the relative accuracy and sensitivity to change of 14 measures commonly used to assess arthritis activity	High correlation between EGA, PGA, and pain scores The PGA along with other measures of disease severity have been shown to be more sensitive to change than laboratory measures (ESR)

*EGA* estimator global assessment, *ESR* erythrocyte sedimentation rate, *HAQ-DI* Health Assessment Questionnaire-Disability Index, *HRQOL* Health Related Quality of Life, *MID* minimally important difference, *PGA* patient global assessment, *SDD* smallest detectable difference, *SF-36* 36-item Short Form Health Survey, SJC swollen joint count, *TJC* tender joint count, *TNF* tumor necrosis factor

In support of the above, in an analysis of the efficiencies to distinguish active treatment from control treatments in clinical trials, among the seven RA core set measures, the highest relative efficiencies were for the physician global estimate followed by PGA and physical function [33]. “Objective” measures of disease, such as acute phase reactants and tender and swollen joint counts, were not superior to “subjective” global estimates of the physician or patient self-report measures of physical function or pain to differentiate active from control treatments. These findings challenge the view that laboratory and clinical examination findings are more robust than patient self-report measures in assessing and monitoring disease progression and treatment response in RA [33].

## Consequences of different wordings/phrasings

The heterogeneity in the wording/phrasing of PGA requires caution when interpreting the results [34]. The DAS28 is one of the most commonly used composite scores in routine clinical practice; the PGA component of the score carries a small weighting of 0.014, which may still result in differences to the overall DAS28 score (the maximum difference being 1.4 when holding the other variables constant and using first a PGA-VAS score of 0 mm, then of 100 mm). French et al. [11] performed a study where DAS28 was calculated in the same patients when using different PGAs. Five different versions of the PGA-VAS were assessed based on: (1) “Feeling” (“How do you feel concerning your arthritis over the last week?”); (2) Disease activity (“How active has your disease been this week?”); (3) “Well-being” (“How has your overall well-being been this week?”); (4) “Best/worst” (“If 0 is the best you have ever been and 100 is the worst you have ever been, where do you think you have been over the last week?”); and (5) “Arthritis impact measurement scales” (AIMS; “Considering all the ways your arthritis affects you….”). All PGA-VAS versions correlated strongly with each other (rho = 0.67–0.87, *p* < 0.0001) [11]. However, when the phrasing of PGA varied there was a difference in DAS28 scores, the largest being 0.63 points. Such differences in score, though small, could have clinical implications, i.e., on the eligibility for biologic disease-modifying anti-rheumatic drugs in countries where access to these drugs is restricted based on strict DAS28 cutoffs.

However, although there are differences at the individual level according to the concepts, wordings/phrasing used, and time period assessed, these differences do not always reflect differences at the group level [11, 12]. Direct comparison between studies is limited due to differences in techniques used to assess the PGA and the population used. Although this has not been explored, it is possible PGA interpretation may be different in clinical trials versus in “clinical practice”, in particular given population selection and the often multiple use of PROs in studies.

Table 4 summarizes the main aspects of the PGA and key messages presented in this review.

**Table 4:** Summary of PGA aspects discussed in this review

Aspects covered	Main findings
Description and practical application	Two different concepts covered: global health versus disease activity
	The wording/phrasing and time-reference used remain unstandardized, leading to differences in interpretation and therefore the responses obtained
	There exist different scales to score PGA
Psychometric properties	Practical, feasible, and non-costly to use in routine clinical practice
	High face validity but its broad concept can lead to difficulties with interpretation
	Good reliability and sensitive to change, making it useful in clinical practice and in research
Consequences of heterogeneity	Differences in interpretation of results
	Impact on DAS28 scoring and therefore the achievement of remission
Elements explaining PGA	RA disease activity as indirectly reflected by inflammation, pain, and functional incapacity (partly due to joint damage) and fatigue explain a large component of the PGA
	Psychological distress can result in higher PGA
	Conflicting evidence exists on the impact of comorbidities on PGA
	Non-RA factors impacting on PGA include demographic characteristics, education, culture, and geographic origin
	Differences in patient understanding and interpretation affect the responses
Discordance between PGA and physician global assessment	More objective measures of disease, e.g., joint counts and acute phase reactants lead to a higher physician global assessment whereas pain and altered quality of life without visible signs of inflammation result in higher PGA
	Patient–physician discordance can affect DAS28 scoring and decision-making, e.g., treatment escalation

## Interpretation of PGA levels for remission

Both DAS28-based remission and ACR/EULAR defined remission criteria incorporate PGA into their scores [2, 35]. ACR/EULAR Boolean-based remission is defined as PGA ≤1 using a 0–10 VAS. Therefore, PGA plays a major role in determining fulfillment of remission criteria in RA [6, 7, 35, 36]. In fact, PGA appears to be often a limiting factor for remission—i.e., in patients with no visible inflammation, remission may not be reached because of PGA. In a study based on the DREAM remission induction cohort, ACR/EULAR remission was present in 20.1 % of the patients. In 108 out of 512 patients, the PGA score was >1 using a 0–10 VAS despite fulfillment of the remaining criteria (TJC28, SJC28, and C-reactive protein in mg/dl ≤1). The specific wording of questions and anchors used for the PGA were: “Considering all of the ways your arthritis affects you, mark “X” on the scale for how well you are doing” (“very well” to “very poor”)” [37]. Similarly, close to half the patients without visible inflammation in the ESPOIR cohort did not achieve ACR/EULAR remission because of PGA levels above 1/10 cm [38]. Thus, near-remission defined as three of the four criteria (PGA excluded) is the most frequent status [36–39]. Many of these patients have a PGA above 1 but still quite low (usual values are around 2/10) [40], perhaps suggesting a need for revising the remission cutoff value for PGA. Another question relates to the phrasing of PGA in the remission criteria: would using the “disease activity” formulation make more sense than using the “global health” formulation? Unpublished results based on the ESPOIR French early arthritis cohort indicate that the disease activity wording will lead to less states of near-remission.

Overall, the high frequency of near-remission raises the question of whether the way remission is defined needs to be better clarified, i.e., should it reflect absence of inflammation alone or absence of inflammation *and* symptoms? The current amalgamation of joint counts and C-reactive protein with PGA indeed leads to some difficulties of interpretation, particularly in cases of near-remission. Furthermore, the predictive validity of near-remission is of clear importance, predicting long-term outcomes of these patients. Our unpublished results indicate near-remission predicts radiographic progression over 3 years in early RA, as well as ACR/EULAR remission, in the ESPOIR cohort, suggesting near-remission is a possible valid and even sufficient predictive outcome in early RA [40, 41]. In the context of treating to target, more work is needed on how to best interpret levels of PGA when aiming for remission.

## What are the elements explaining PGA?

PGA is a wide-reaching measure which may mean different things for different people. Data are available on the main drivers of PGA at the group level. PGA reflects both disease activity and other factors. Given PGA is assessed to provide information additional to joint counts or acute phase reactants, it is expected there might be some but not complete overlap.

### PGA is explained by RA disease activity

Disease activity (i.e., RA inflammatory status) explains a large part of PGA. Pain is a major cause of distress in patients with RA and this, along with joint damage, is among the important aspects/domains of RA that affect patients’ lives and will contribute to how the PGA is scored. Most studies indeed support that pain and functional incapacity (evaluated by HAQ), are the most important drivers of PGA, and these outcomes are indirectly reflecting RA disease activity. Furthermore, fatigue plays a role and has also been found to be an important determinant of PGA [13]. However, joint counts and acute phase reactants are not strong drivers of PGA [12, 36]. In several studies pain is the single main driver of PGA and may explain up to 75 % of the PGA result, whether the concept is global health or disease activity [11, 13]. This high contribution from pain is multifactorial but strongly related to the inflammatory status [42]. This probably contributes to its high responsiveness in trials. We should recognize, however, that pain itself is multifactorial.

With regard to concepts underlying PGA, as expected the disease activity-PGA is more related to inflammation than the global health-PGA. Data from the Quantitative Standard Monitoring of Patients with RA (QUEST-RA) study support that the PGA is explained by different drivers depending on the wording used [12].

### PGA is explained by non-RA factors

Over and above disease activity, PGA is affected by other factors, including RA-related factors such as structural damage and non-RA factors such as demographic characteristics, education level, and perhaps culture and geographic origin (Table 1) [36, 43]. Furthermore, interpretation of the question by the patient may depend on duration of disease through a “response shift” resulting from better familiarity with symptoms and changes in patient expectations, as mentioned above.

In the QUEST-RA study, psychological distress was an important driver of PGA and was influenced by the different wording used for the PGA: it was driving more importantly global health than the disease activity wording [13]. There is conflicting evidence on the impact of comorbidities on PGA: a cross-sectional study of US Hispanics with RA showed no association between comorbidities, including depression and fibromyalgia, and PGA [44]. In contrast, based on a study of 50 female patients with RA, in those with co-existing fibromyalgia, significantly higher subjective items, including the PGA (using global health wording) were noted [45]. Differences in the study population and design, as well as the collection and recording of comorbidity data, could account for the variations seen between studies.

## Discordance between PGA and physician global assessment

It is interesting to compare PGA to another global, “gestalt” assessment of disease, which is the physician global assessment. Physician global assessment is a well-validated outcome which is recognized as part of the RA core set [3]. Evidence suggests that discordance exists between patient and physician assessment of RA disease activity, with studies consistently showing that PGA is very often scored higher than physician global assessment [13, 43, 46–49]. Discordance in most studies is defined as a difference of ≥3/10 points between the PGA and physician global assessment [12]. The prevalence of discordance using this definition was found to be around 43 % in a recent meta-analysis, indicating a different understanding or perspective of the same general concept [48].

What studies have shown to date is that variables that are important to patients are not the same as those valued by physicians as reflecting disease activity (Table 5). Generally, more objective measures of disease, e.g., joint counts and elevated acute phase reactants, lead to a higher physician global assessment whereas pain and altered quality of life without visible signs of inflammation, but also comorbidities and psychological distress, will lead to higher PGA (Table 5) [50]. In such cases of discordance, it is important to discuss the patient’s psychological status as well as personal life factors since the solutions will not always lie in immunosuppressive drugs but rather might depend on non-pharmacological interventions. This discordance may act as a clue to the presence of non-disease severity factors influencing the PGA.

**Table 5:** Discordance between PGA and estimator global assessment and associated factors

Study group	Study description	Patient number (n)	Discordance between PGA and EGA	Factors associated with discordance
Desthieux et al. 2016 [55]	Systematic literature review and meta-analysis	11,879 (12 studies)	Frequency of discordance >2.7 cm (weighted mean cutoff): 44.9 % PGA > EGA: 79.1 % PGA < EGA: 20.9 %	Drivers of global assessment: PGA: pain (the most frequent driver of PGA, significant in eight studies [100 % of studies analyzing this driver of PGA]), functional incapacity, fatigue EGA: swollen and tender joint counts, acute phase reactants Drivers of discordance: Depressive symptoms, health literacy
Davis et al. 2014 [56]	Consecutive RA patients	127	Frequency of discordance: PGA > EGA: 16.5 % PGA < EGA: 10.2 %	PGA > EGA: pain, fatigue, HAQ disability, poor health related quality of life on the SF-36 PGA < EGA: higher numbers of swollen joints, positive rheumatoid factor and lower pain; better overall physical and mental health
Khan et al. 2012 [13]	Patients from the multi-national Quantitative Standard Monitoring of Patients with RA (QUEST-RA) database	7028	Mean PGA 4.0 ± 2.7 cm; EGA 2.9 ± 2.4 cm Frequency of discordance >2 cm: PGA > EGA: 30 % PGA < EGA: 6.6 %	PGA > EGA: higher age; higher scores of pain, fatigue, HAQ, and morning stiffness PGA < EGA: higher SJC, TJC, ESR; lower fatigue score
Barton et al. 2010 [48]	Multi-site observational cohort with RA adults consecutively enrolled from two outpatient clinics in the US	223	Mean PGA 4.3 ± 2.6 cm; EGA 3.1 ± 2.1 cm Frequency of discordance >2.5 cm: PGA > EGA: 31 % PGA < EGA: 5 %	PGA > EGA: higher HAQ score; lower SJC; greater depressive symptoms
Nicolau G et al. 2004 [43]	Single center cohort of RA patients in Brazil	80	Frequency of discordance ≥1 cm: PGA > EGA: 44 % PGA < EGA: 28 % Frequency of discordance ≥3 cm PGA > EGA: 24 % PGA < EGA: 9 %	PGA > EGA: higher pain and HAQ scores and tendency for higher number of comorbid conditions
Studenic et al. [42]	Single center observational cohort of RA patients initiating MTX in Austria	646	Mean PGA 3.9 ± 2.7 cm; EGA 2.3 ± 2.1 cm Frequency of discordance ≥0.5 cm: PGA > EGA: 61 % PGA < EGA: 15 %	PGA > EGA: higher pain and lower SJC

*EGA* estimator global assessment, *ESR* erythrocyte sedimentation rate, *HAQ-DI* Health Assessment Questionnaire-Damage Index, *MID* minimally important difference, *PGA* patient global assessment, *SDD* smallest detectable difference, *TJC* tender joint count, *SF-36* 36-item Short Form Health Survey

## Discussion

The increasing emphasis on the patients’ perspective of health in considering priorities and making treatment choices has resulted in PROs being a core part of routine assessment of disease in RA and also an end-point in clinical trials and observational studies. Recent guidelines are characterized by a shift from the traditional approach of physician-led physical examination and investigations such as laboratory tests and radiographs (the “biomedical model”) to a more patient-centered approach to care [51]. PGA is one of the most commonly captured and reported PROs, mainly due to its simplicity and its feasibility in both clinical practice and registers as well as in clinical trial settings. It is strongly correlated with other self-reported outcomes and carries important patient information. Therefore, despite the controversy regarding the value of PROs including the PGA, these represent the only way to assess some of the aspects related to RA, for example, symptoms, justifying that clinician-reported outcomes and PROs should be considered as complementary to each other [52].

The lack of homogeneity in the concepts, wordings/phrasings, and time period assessed by PGA threatens the validity of PGA since it may lead to modified responses resulting from the diversity of formulations [15]. Such diversity can influence clinical and treatment decision-making, highlighting the importance of standardizing (where appropriate) and validating the question phrasing as part of capturing information on PGA. We suggest that emphasis is placed on reducing heterogeneity in wording/phrasing and time period of the PGA in order to enable more uniform capture (and hence interpretation) of information across clinical and research settings.

The discordance between PGA and physician global assessment demonstrated in many studies to date suggests that perceptions of disease activity by patients may be influenced by different factors, resulting in different aspects of disease being measured. Such discordance can negatively influence medical care, adherence to treatment, and disease outcomes. Despite this, the use of PROs such as PGA is particularly informative, bringing additional information and perspective, especially given the observed discordance of assessment between physicians and patients [52]. Like other PROs, it is of particular value when changes in clinical measurements or laboratory or radiographic outcomes may not translate into meaningful benefits for patients. In particular, the ease of use and feasibility of PGA, with little or no training of patients required to complete it, means that it can be easily incorporated into busy clinical settings. However, it is important that this contribution of patients to disease activity scores via the PGA is evaluated in a standardized way.

We feel the lack of a standardized definition on the concept, wording/phrasing, and time period assessed as part of PGA represents one of its weaknesses. This does not preclude the possibility of having more than one version of PGA; however, it requires clarity with regard to what version is selected and for which purpose (e.g., PGA for disease activity or PGA for global health). We advocate the use of a homogeneous wording in a specific context, e.g., for repeated measures it is important to use the same wording/phrasing. This is particularly important in routine clinical practice since it may lead to incorrect interpretations regarding, for example, response to treatment.

In terms of practical recommendations, the suggestion of the authors is that phrasing of PGA may at this stage be proposed as capturing (1) either *global health* or *disease activity* which is (2) *related to arthritis* and captured by the reference period of (3) *today*/*this point in time*. Examples would be the formulation proposed in the RAPID 3 score for global health or the one proposed in the EULAR/ACR remission criteria for disease activity (Table 6) [37].

**Table 6:** Proposals of wording/phrasing in view of homogenizing PGA

Concept	Global health	Disease activity
Wording/phrasing	“Considering all the ways in which illness and health conditions may affect you at this time, please make a mark below to show how you are doing”	“Considering all the ways your arthritis has affected you, how do you feel your arthritis is today?”
Scoring	0–100 VAS or 0–10 NRS	0–100 VAS or 0–10 NRS
Anchors	Very well–very poorly	Inactive–very active

The choice between the two concepts will depend on the objective of the measurement of the PGA. The global health question gives more holistic information on patient status since it includes to a wider extent elements such as comorbidities and psychological distress. The disease activity-PGA is more in line with more objective measures of disease and assesses more closely the inflammatory burden.

## Conclusions

PGA is a key outcome measure in RA with clear validity and usefulness. However, the lack of a “gold standard” in terms of its wording/phrasing and time period assessed necessitates more research into this field in order to avoid pitfalls in interpretation and, consequently, in the achievement of treatment targets. In this review, we propose homogenized wordings which may be considered for future studies. Importantly, a clear understanding of what PGA measures and potential sources of variation in its reporting is key to accurate interpretation. Furthermore, this review gives insights into factors associated with and affecting PGA—for example, its close association with pain, mood, and fatigue and issues around discordance with physician global assessment. This may be informative in guiding interventions to improve care and overall quality of life for RA patients.

## Abbreviations

ACR: American College of Rheumatology
DAS: Disease Activity Score
DAS28: 28-joint count Disease Activity Score
DREAM: Dutch Rheumatoid Arthritis Monitoring (registry)
EGA: Evaluated global assessment
ESPOIR: Etude et Suivi des POlyarthrites Indifferenciées Récentes (cohort)
ESR: Erythrocyte sedimentation rate
EULAR: European League Against Rheumatism
HAQ: Health Assessment Questionnaire
NRS: Numeric rating scale
PGA: Patient global assessment
PROs: Patient reported outcomes
QUEST-RA: Quantitative Standard Monitoring of Patients with Rheumatoid Arthritis
RA: Rheumatoid arthritis
RAPID 3: Routine Assessment of Patient Index Data
SJC: Swollen joint count
TJC: Tender joint count
VAS: Visual analogue scale
